# Inducible nitric oxide synthase enhances disease aggressiveness in pancreatic cancer

**DOI:** 10.18632/oncotarget.10323

**Published:** 2016-06-29

**Authors:** Jian Wang, Peijun He, Matthias Gaida, Shouhui Yang, Aaron J. Schetter, Jochen Gaedcke, B. Michael Ghadimi, Thomas Ried, Harris Yfantis, Dong Lee, Jonathan M. Weiss, Jimmy Stauffer, Nader Hanna, H. Richard Alexander, S. Perwez Hussain

**Affiliations:** ^1^ Pancreatic Cancer Unit, Laboratory of Human Carcinogenesis, CCR, NCI, Bethesda, MD, USA; ^2^ Institute of Pathology, University Hospital of Heidelberg, Heidelberg, Germany; ^3^ US Food and Drug Administration, Silver spring, MD, USA; ^4^ Department of General, Visceral and Pediatric Surgery, University Medicine, Göttingen, Germany; ^5^ Genetics Branch, CCR, NCI, Baltimore Veterans Affairs Medical Center, Baltimore, MD, USA; ^6^ Pathology and Laboratory Medicine, Baltimore Veterans Affairs Medical Center, Baltimore, MD, USA; ^7^ Cancer and Inflammation Program, CCR, NCI Frederick, MD, USA; ^8^ Laboratory of Cell and Developmental Signaling, NCI Frederick, MD, USA; ^9^ Division of Surgical Oncology, University of Maryland School of Medicine, Baltimore, MD, USA

**Keywords:** NOS2, NO, PDAC, KPC mouse model

## Abstract

Pancreatic cancer is one of the most lethal malignancies and is refractory to the available treatments. Pancreatic ductal adenocarcinoma (PDAC) expresses high level of inducible nitric oxide synthase (NOS2), which causes sustained production of nitric oxide (NO). We tested the hypothesis that an aberrantly increased NO-release enhances the development and progression of PDAC. Enhanced NOS2 expression in tumors significantly associated with poor survival in PDAC patients (*N* = 107) with validation in independent cohorts. We then genetically targeted NOS2 in an autochthonous mouse model of PDAC to examine the effect of NOS2-deficiency on disease progression and survival. Genetic ablation of NOS2 significantly prolonged survival and reduced tumor severity in *LSL-Kras*^G12D/+^; *LSL-Trp53^R172H/+^*; *Pdx-1-Cre* (KPC) mice. Primary tumor cells isolated from NOS2-deficient KPC (NKPC) mice showed decreased proliferation and invasiveness as compared to those from KPC mice. Furthermore, NKPC tumors showed reduced expression of pERK, a diminished inactivation of Forkhead box transcription factor O (FOXO3), a tumor suppressor, and a decrease in the expression of oncomir-21, when compared with tumors in KPC mice. Taken together, these findings showed that NOS2 is a predictor of prognosis in early stage, resected PDAC patients, and provide proof-of-principle that targeting NOS2 may have potential therapeutic value in this lethal malignancy.

## INTRODUCTION

Pancreatic cancer is a highly lethal malignancy with median survival of less than 6 months, and ranks as the 4th leading cause of cancer-related death in the United States. An estimated 53,070 new cases of pancreatic cancer will be diagnosed and 41,780 deaths occur in 2016 alone due to this malignancy [1]. Pancreatic ductal adenocarcinoma (PDAC) is the most common form, which accounts for more than 90% of all the pancreatic cancer cases. The lack of early detection markers and ineffective treatment in advanced disease have led to a consistent rise in both incidence and mortality in pancreatic cancer, which is estimated to become the second leading cause of cancer-related death by 2030 [2]. Therefore, identification and pre-clinical/clinical assessment of novel therapeutic targets are urgently needed to improve disease outcome in patients with PDAC. The recent advances in our understanding of the complex pancreatic cancer biology and tumor architecture have started to emerge as opportunities to design improved treatments.

PDAC has a highly inflammatory tumor microenvironment, and inflammatory mediators produced by both tumor and stromal cells are implicated in the development and progression of this malignancy [3]. NO is a free-radical and an important mediator of immune and inflammatory responses, in addition to its role in critical biological processes including vasodilation and neurotransmission. However, there are evidences implicating a role of NO in the development and progression of cancer [4–9]. NO is produced by a family of nitric oxide synthase (NOS) enzymes, which includes NOS1 (neuronal NOS), NOS2 (inducible NOS) and NOS3 (endothelial NOS). Whereas, NOS1 and NOS3 are the constitutive isoforms and produce a small amount of NO, NOS2 is an inducible isoform and produces a higher and sustained level of NO in micromolar range in response to inflammatory stimuli. Therefore, NOS2 is primarily responsible for an enhanced level of NO production [4]. The exact role of NO in pancreatic cancer is not clearly defined, and is somewhat contradictory. For example, highly aggressive property of pancreatic cancer cell line Panc02-H7, generated through successive *in vivo* selection procedure, was associated with a lower NOS2 expression [10] and NO treatment using NO-donor drug inhibited proliferation and invasion in pancreatic cancer cell line [11]. In an earlier study the negative or positive NOS2 immunohistochemical expression did not show any association with prognosis in PDAC [12]. However, genetic ablation of NOS3 or endothelial NOS inhibited the development of precursor lesions or pancreatic intraepithelial neoplasia (PanINs) in a genetically engineered mouse model of pancreatic cancer [9]. A higher expression of NOS2 is reported in PDAC [13]. In this study, we first determined the clinical relevance of NOS2 by assessing its association with the survival of a large cohort of early stage, resected patients with PDAC, and then delineated the role of NOS2/NO signaling in the development and progression of PDAC, using a genetic strategy in the KPC mouse model of PDAC. We hypothesized that NOS2/NO signaling enhances the development and progression of PDAC and is a potential therapeutic target. To test this hypothesis, we first examined the tumors from a large cohort (*N* = 107) of resected PDAC patients and found that an increased NOS2 gene expression level was associated with poorer survival in these patients, which we validated in two additional independent cohorts. Secondly, using a genetic strategy we showed that NOS2-deficiency significantly enhanced the survival in KPC mouse model of pancreatic cancer, which closely recapitulates the development and progression of human PDAC. Taken together, these findings provide proof-of-principle that targeting NOS2/NO signaling may be a useful strategy for improving survival in patients with PDAC and should be pursued in future clinical trials.

## RESULTS

### A higher NOS2 expression is associated with poor survival in patients with PDAC

**Figure 1:**
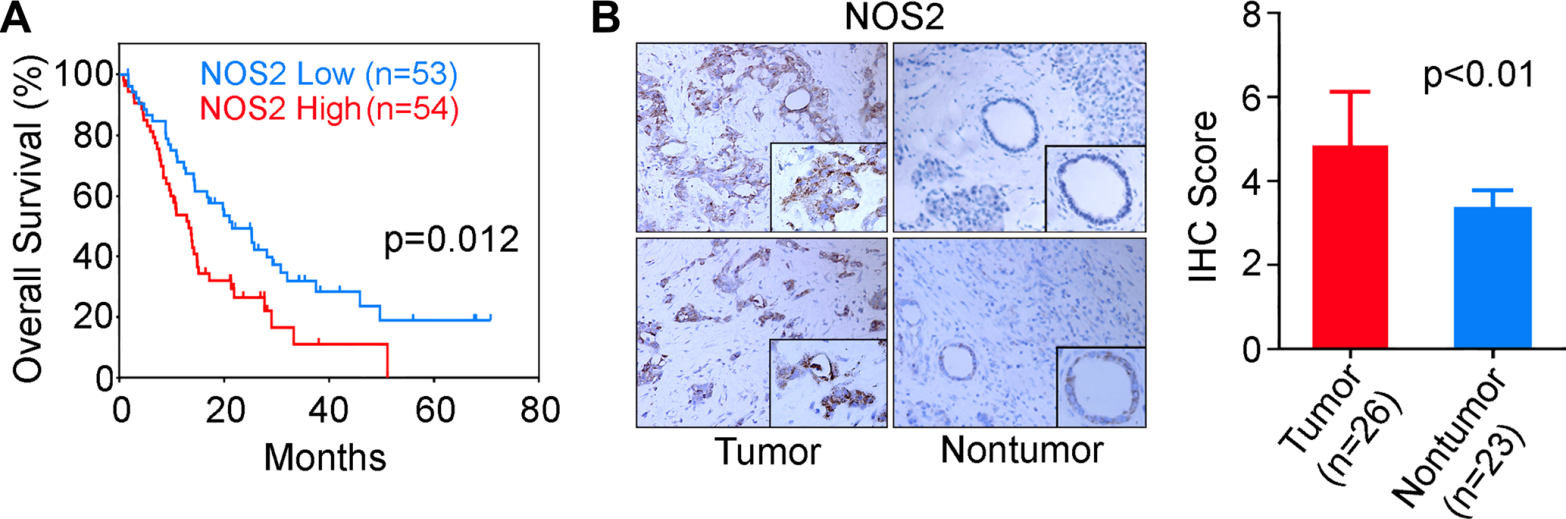
Higher expression of NOS2 is associated with poor survival in patients with PDAC (**A**) Kaplan-Meier survival curve was plotted based on the median value of NOS2 expression, as determined by qRT-PCR in tumors from PDAC patients. Patients with the NOS2 expression above the median value were treated as high NOS2 group, and patients with the NOS2 expression below the median value, as low NOS2 group. Log-rank test was used to determine the significance of difference in survival between the high and low NOS2 groups. (**B**) Immunohistochemical (IHC) staining showing NOS2 expression in tumors and adjacent nontumor tissue in PDAC cases.

To assess the clinical relevance of NOS2/NO signaling in the patients with PDAC, we first examined the NOS2 expression by qRT-PCR in tumors from 107 resected cases. Patients were then divided into NOS2-high and NOS2-low groups based on the median value of NOS2 expression. Patients with the tumor NOS2 expression above the median were defined as NOS2-high group, and the patients with the NOS2 expression lower than the median value constituted NOS2-low group. Kaplan-Meier analysis showed that patients with a higher NOS2 had significantly poorer survival as compared to the patients with lower NOS2 expression in resected tumors (Log-rank test *p* = 0.012) (Figure 1A). Additionally, a higher NOS2 in tumors predicted poor prognosis by both univariable (hazard ratio = 1.73, 95% CI = 1.09–2.75, *p* = 0.020) and multivariable (hazard ratio = 1.75, 95% CI = 1.10–2.79, *p* = 0.018) Cox-regression analyses (Supplementary Table S1). Furthermore, consistent with the earlier report [13], the tumors in our patient cohort showed a significantly higher NOS2 expression as compared to the adjacent nontumor pancreas (*P* < 0.01, *N* = 26) (Figure 1B). We then validated our findings in publically available datasets. Analysis of the pancreatic cancer cohorts in The Cancer Genome Atlas (TCGA) and Oncomine database (https://www.oncomine.org/resource/login.html) [Collision cohort [14]] also showed that a higher tumor NOS2 expression associates with poorer survival in patients with PDAC (Kaplan-Meier analysis, Log-rank test *p* = 0.041 and *p* = 0.022) (Supplementary Figure S1), which is consistent with the findings in our test cohort of 107 resected patients. These findings showed that NOS2 is a candidate prognostic marker in early stage PDAC patients undergoing surgical resection, and NOS2/NO signaling may play a role in enhanced disease progression.

### NOS2-deficiency enhanced survival and reduced tumor severity in genetically engineered mouse model of pancreatic cancer (KPC mouse)

**Figure 2:**
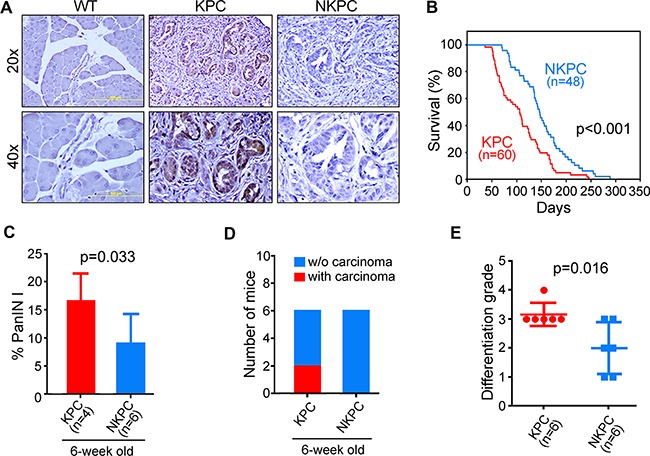
NOS2-deficiency in KPC mice resulted in better survival and less tumor severity (**A**) NOS2 expression by IHC in pancreas from KPC, NKPC and the wild-type (WT) littermates mice. NOS2 expression was elevated in KPC compared to WT mice, whereas the expression of NOS2 protein was not detectable in NKPC pancreas. (**B**) Survival of the KPC and NKPC mice was determined by Kaplan-Meier survival analysis (Log-rank). NOS2-deficiency significantly increased survival in NKPC mice (*N* = 48) as compared with KPC mice *(N* = 60). (**C**) One hundred ducts were examined using H/E sections of each 6-week old mouse pancreas from the two groups. On average, around 17% of the ducts in KPC mouse pancreas showed panIN-1 lesions, compared to less than 10% of PanIN-1 lesions among NKPC pancreatic ducts. (**D**) Pathological examination revealed that 2 out 6 KPC mice developed carcinomas compared to its complete absence in NKPC mice by 6 week of age. (**E**) The differentiation grade of tumors from the two groups was evaluated. Tumors in NKPC mice showed overall better differentiation as compared with KPC mice.

To elucidate the role of NOS2/NO signaling in PDAC, we took the genetic strategy by deleting the NOS2 gene from the KPC mouse model of PDAC, as described in materials and methods, and generated NOS2-deficient KPC mice, which we termed as NKPC. KPC mice with the pancreas-specific activation of mutant-KRAS and -TP53 mirrors the key histopathological and clinical characteristics associated with the development and progression of human PDAC. Pancreatic tumors in KPC mice expressed a high level of NOS2 protein, which, as expected, is undetectable in tumors from NKPC mice (Figure 2A). Kaplan-Meier analysis showed that NKPC mice survived significantly longer than KPC mice (Log-Rank test, *p* < 0.001) (Figure 2B). To gain further insights into the effect of NOS2/NO signaling on pancreatic tumorigenesis, some of the KPC and NKPC mice were sacrificed at an early age of 6 week. Although only 6 mice were sacrificed at 6 weeks of age, examination of 100 randomly selected pancreatic ducts in the pancreas of each mouse showed a significantly lower percentage of precursor-lesion, pancreatic intraepithelial neoplasia-1 (PanIN1), in NKPC mice as compared with KPC mice (Figure 2C). Additionally, 2 out of 6 KPC mice showed the presence of carcinoma in their pancreas at 6 week of age, in contrast to the absence of any carcinoma in the pancreas of NKPC mice at this age (Figure 2D). Furthermore, the PDAC in KPC mice were of higher differentiation grade as compared to that of NKPC mice (Figure 2E) (Supplementary Figure S2). These findings showed that NOS2-deficiency resulted in increased survival in mice with lethal PDAC, and reduced tumor severity.

### NOS2-deficiency inhibited proliferation, migration and invasion of primary tumor cells and enhanced apoptosis in tumors from NKPC mice

**Figure 3:**
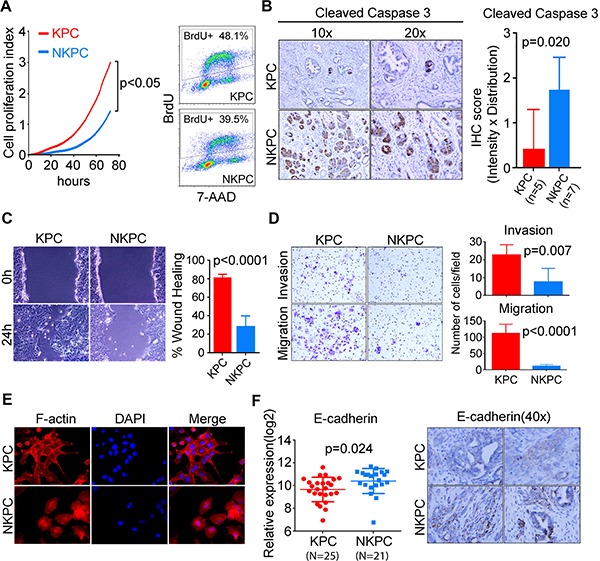
NOS2-deficiency reduced proliferation and invasiveness in primary tumor cells and increased apoptosis in tumors from NKPC mice (**A**) Proliferation of the primary tumor cells from KPC and NKPC mice was determined using Xcelligence method and by BrdU assay, showing that NOS2-deficiency significantly reduced proliferation. (**B**) An enhanced apoptotic cell death in NKPC tumors, determined by cleaved Caspase 3 staining, as compared with tumors in KPC mice. IHC scoring was done by multiplying the intensity and distribution of the staining and the results were analyzed by Student's *t*-test. (**C**) Wound healing assay showing a reduced migration of primary tumor cells from NKPC mice as compared with KPC mice. (**D**) Metastatic potential of primary tumor cells were also evaluated using transwell assay. NKPC tumor cells showed a significantly reduced migration and invasion as compared with KPC tumor cells. (**E**) F-actin in KPC and NKPC primary tumor cells were visualized by Rhodamine-conjugated Phalloidin staining. Tumor cells with or without NOS2 showed distinct morphology, with filopodia-like protrusion, a typical feature facilitating cell migration, only observed in KPC primary tumor cells but not in NKPC tumor cells. (**F**) An increased expression of E-cadherin in tumors from NKPC (*N* = 21), compared to KPC mice (*N* = 25), as determined by qRT-PCR. All the experiments were repeated 3 times. Also shown is a representative picture of E-cadherin expression by immunohistochemistry.

To examine the effect of NOS2/NO signaling on the tumorigenic potential of pancreatic cancer cells, we isolated primary tumor cells from KPC and NKPC mice. Primary tumor cells from NKPC mice showed a lower proliferation index as compared to KPC mice as determined by real-time and dynamic monitoring of cell proliferation using Xcelligence system (Figure 3A, Supplementary Figure S3). The reduced proliferation of primary tumor cells in NKPC mice was also confirmed by using BrdU incorporation assay (Figure 3A). Suppression of apoptosis is one of the critical events in tumor initiation, progression and metastasis. NO has been implicated in modulating the apoptotic pathways and may have pro- or anti-apoptotic function depending on a number of biological factors including the amount of NO and the cellular context, presence of metals and interaction with other reactive oxygen species [reviewed in [15]]. Immunohistochemical staining of cleaved Caspase-3 indicated a significantly increased apoptosis in pancreatic tumors from NKPC mice as compared with KPC tumors (Figure 3B). Next we determined if the NOS2-deficiency affected the migratory and invasive properties of primary tumor cells. Primary tumor cells from NKPC mice showed a significant reduction in both migration and invasion properties as compared with KPC primary tumor cells (Figure 3C–3D). Consistent with these findings, the primary tumor cells from KPC mice presented a distinct morphological feature with abundant filopodia-like protrusions, which is described to aid in cell migration (Figure 3E), in contrast to the NKPC primary tumor cells, as shown by the staining of F-actin using florescence labeled phalloidin. Additionally, a higher expression of E-cadherin, a marker of EMT phenotype, was found in primary tumor cells by qRT-PCR and could also be visualized by immunohistochemical staining in tumor tissue from NKPC as compared to KPC mice (Figure 3F). Recently, an actin bundling protein, Fascin, has been described to aid in the formation of filopodia, resulting in an enhanced invasiveness of PDAC cells [16]. We found a significantly reduced expression of Fascin in primary tumor cells from NKPC as compared with KPC mice, which is consistent with the reduced migration and invasion phenotypes in NKPC tumor cells (Supplementary Figure S4). These findings showed that NOS2/NO signaling enhances the tumorigenic properties of pancreatic cancer cells in the KPC mouse model of PDAC.

### NOS2-deficiency reduced tumor macrophage infiltration, chemokine ligand-2/MCP1 and mir-21 expression in KPC mice

**Figure 4:**
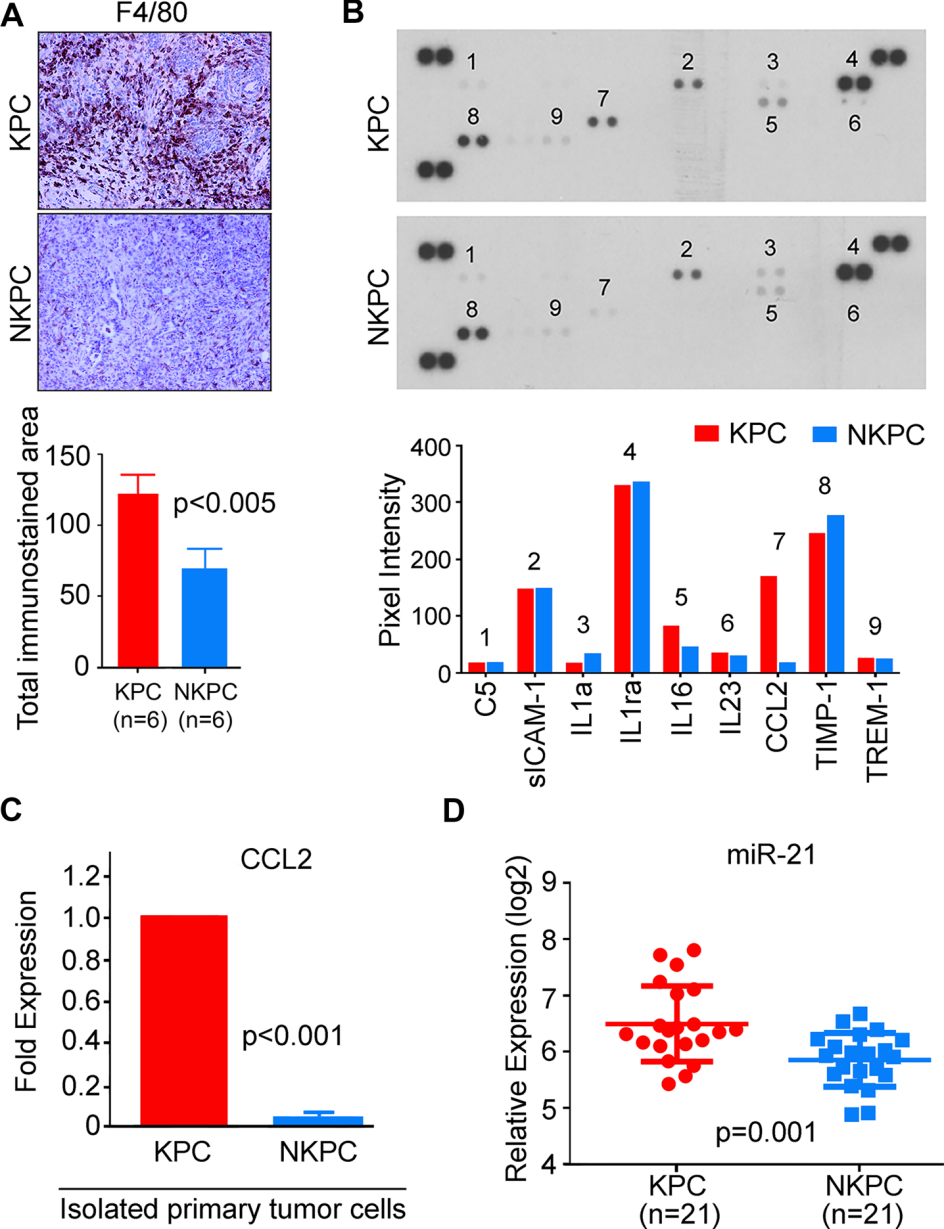
NOS2-deficiency reduced inflammatory response in KPC tumors (**A**) NOS2 depletion resulted in reduced macrophage infiltration in tumors. IHC staining of macrophage marker F4/80, showing an increased macrophage infiltration in KPC tumors, as compared with tumors from NKPC mice. Quantification of IHC staining was performed by computerized screening of total stained area on each slide as described earlier [44]. The average stained area is shown in histogram and analyzed by Student's *t*-test. Data were collected from 6 mice in each group. (**B**) Cytokine levels within pancreatic tumor tissues were examined by mouse cytokine array, and the quantitation of visible dots was performed by Quantity One software (Biorad, Hercules, CA). As shown, a significant reduction in CCL2 was observed in NKPC tumors compared to KPC tumors. Analysis was conducted in the pooled serum samples from 10 mice in each group. (**C**) A significantly lower CCL2 expression in NKPC as compared to KPC primary tumor cells, as determined by qRT-PCR. (**D**) A decreased expression of miR-21, as determined by qRT-PCR, in NKPC (*N* = 21) tumors as compared with tumors in KPC (*N* = 21) mice. Statistical significance was evaluated by using Student's *t*-test. Unless, indicated otherwise, experiments were repeated at least 3 times.

Inflammation contributes to the development and progression of PDAC. Similar to the characteristic desmoplastic stroma in human PDAC, a highly reactive, inflammatory stroma is present in the pancreatic tumors of KPC mice. Because NO is a known mediator of inflammation, we examined, if NOS2-deficiency affected the inflammatory microenvironment of PDAC in KPC mice. Compared to tumors in KPC mice, NKPC tumors showed significantly reduced macrophages infiltration, as determined by immunohistochemical analysis of F4/80, a murine macrophage marker (Figure 4A, Supplementary Figures S5, S6). Macrophage infiltration has been reported previously as a negative prognostic marker for PDAC [17–19]. Furthermore, cytokines and chemokines play important role in inflammatory responses and have been implicated in pancreatic tumor development and progression. Several feedback regulatory circuits have been described between nitric oxide and cytokines that are produced by tumor as well as inflammatory cells. Therefore, next, we determined the level of cytokines/chemokines in tumors from KPC and NKPC mice, using a mouse cytokine array panel containing 40 different cytokines. A marked decrease in the expression of chemokine ligand 2 (CCL2), also known as monocyte chemoattractant protein-1 (MCP1) was found in NKPC tumors as compared with tumors in KPC mice (Figure 4B). Consistent with these findings, qRT-PCR analysis of primary tumor cells also showed a lower expression of CCL2 in NKPC as compared with KPC mice (Figure 4C). Inflammatory mediators including cytokines, regulate miRNAs. Several microRNAs are implicated in inflammation-associated carcinogenesis, and genetic ablation of NOS2 in a genetically engineered mouse model of KRAS-induced lung cancer reduced the expression of oncomir-21 [20, 21]. Additionally, an increased expression of mir-21 is associated with poor prognosis in pancreatic cancer [22]. Therefore, we tested the hypothesis that NOS2/NO signaling alters mir-21 expression in PDAC. qRT-PCR analysis revealed a significant decrease in the expression of mir-21 in NKPC as compared to tumors from KPC mice (Figure 4D). Taken together, these findings show that NOS2-deficiency decreases inflammation and mir-21 expression in PDAC of KPC mice.

### NOS2-deficiency inhibited ERK-signaling and phosphorylation of FOXO3

**Figure 5:**
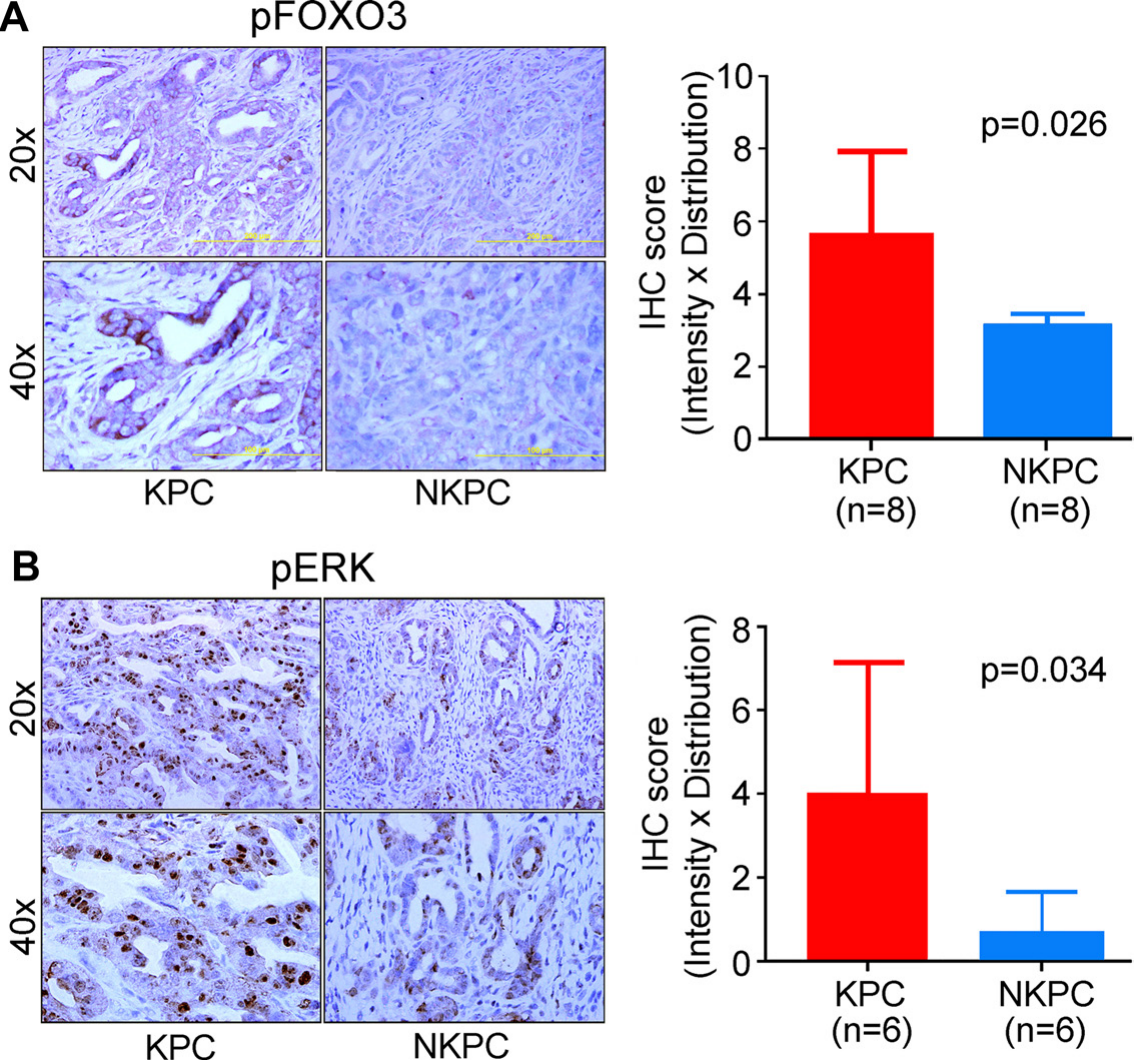
NOS2-deficiency inhibited ERK signaling and phosphorylation of FOXO3 Immunohistochemical staining, showing a reduced expression of pFOXO3 (**A**), and pERK (**B**) in pancreatic tumors from NKPC, as compared with KPC mice. The staining was evaluated by multiplying intensity by distribution score as described earlier [45]. Histograms show the quantitation of IHC staining and the results were analyzed by Student's *t*-test.

**Figure 6:**
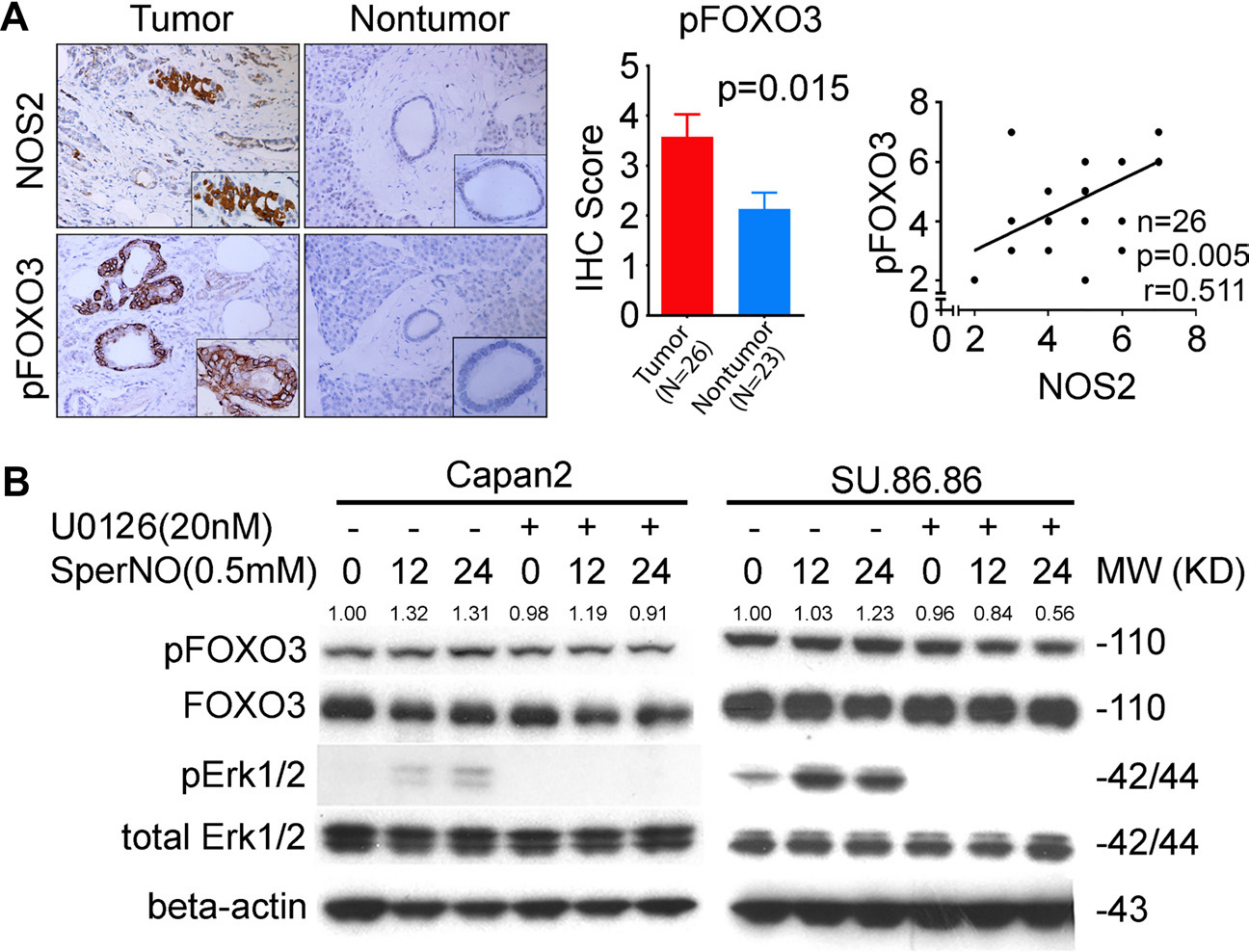
Nitric oxide induces ERK signaling and the subsequent phosphorylation of FOXO3 in human pancreatic cancer (**A**) Immunohistochemical staining showing an increased expression of NOS2 and pFOXO3 in tumors as compared with adjacent nontumor pancreatic ducts from patients with PDAC. NOS2 and pFOXO3 showed a positive correlation in human PDAC as analyzed using Pearson correlation. (**B**) An increase in pERK and pFOXO3 was found following NO treatment in pancreatic cancer cell lines. Human PDAC cell lines Capan2 and SU.86.86 were treated with nitric oxide donor, Sper/NO at 0.5 mM concentration for up to 24 hours, with or without MEK inhibitor, U0126 (20 nM), the phosphorylated and total FOXO3 and ERK proteins were blotted with corresponding antibodies. Levels of pFOXO3 were quantitated relative to total FOXO.

FOXO (Forkhead box-O) is a member of the forkhead family of transcription factors, which regulates several critical functions responsible for maintaining cellular homeostasis, including apoptosis, DNA repair, cell cycle arrest and oxidative stress, and is reported to have tumor suppressive function [23–25]. FOXO is negatively regulated through phosphorylation by PI3K/AKT and ERK-signaling pathway, which leads to its nuclear exclusion [26, 27]. Inactivation of FOXO is reported in cancer [25]. NO can activate both PI3K/AKT and ERK-signaling [28, 29]. Therefore, we hypothesized that NOS2/NO signaling may result in the increased phosphorylation and, therefore, inactivation of FOXO. Immunohistochemical analysis showed an increase in phosphorylated FOXO3 (pFOXO3) in tumors from KPC as compared with the NKPC mice (Figure 5A). As expected, the phosphorylated FOXO3 was exclusively located in cytoplasm of tumor cells. Additionally, KPC tumors also showed an increase in phosphorylated ERK (pERK) as compared with tumors in NKPC mice (Figure 5B). These findings indicate a potential contribution of NO-ERK-FOXO3 signaling in pancreatic tumorigenesis in KPC mice, and the reduced phosphorylation of FOXO, in the context of NOS2-deficiency, may contribute to the reduced tumor severity and increased survival in NKPC as compared with KPC mice. To further determine the relevance of NO mediated inactivation of FOXO3 in human PDAC, we examined the expression of pFOXO3 and NOS2 in tumors and adjacent nontumor tissue from resected patients. An increased expression of both NOS2 as well as pFOXO3 was found in tumors, when compared with adjacent nontumor pancreatic ducts (Figure 6A). Importantly, a positive correlation was found between pFOXO3 and NOS2 in PDAC (Figure 6A). To further delineate, if NO actually induces the phosphorylation of FOXO3, we treated the human pancreatic cancer cell lines, Capan2 and SU.86.86 with a nitric oxide donor, Spermine/NONOate (Sper/NO), which resulted in an increased expression of both pERK and pFOXO3 (Figure 6B). However, inhibition of ERK activation by simultaneous treatment with U0216, a MEK inhibitor, abolished the effect of NO on the phosphorylation of FOXO3 in these cell lines. These findings showed that NO enhances the phosphorylation of FOXO3 through the activation of ERK-signaling pathway and that NO-ERK-FOXO signaling may play a role in the development and progression of human PDAC.

## DISCUSSION

Pancreatic cancer is mostly diagnosed at an advanced stage and is highly resistant to the available treatments. A small number of patients, detected at an early stage, undergoes surgical resection with curable intent, however, a large percentage of resected patients show recurrence within 2 years. Therefore, identification, validation, and preclinical and clinical assessment of effective therapeutic targets are urgently needed to improve patient outcome in this lethal malignancy. An increased and sustained production of NO is implicated in the development and progression of cancer. In this study, we tested the hypothesis that NOS2/NO signaling enhances pancreatic cancer progression and is a potential therapeutic target. We report that an increased NOS2 expression is associated with poor survival in early stage patients with resected PDAC, with validation in independent cohorts. Additionally, genetic ablation of NOS2 in a genetically engineered mouse model of pancreatic cancer significantly increases survival, thus, providing proof-of-concept that therapies targeting NOS2 may improve survival in patients with PDAC.

The role of NO in tumorigenesis is highly complex and both pro- and anti-neoplastic functions have been reported, which largely depends on the amount of NO, cell types, cellular microenvironment, its interaction with other reactive species and presence of metals in the neoplastic cells. For example, genetic deletion of NOS2 in P53-deficient mice can either suppress or enhance lymphomagenesis depending on the inflammatory microenvironment [30, 31]. Furthermore, NOS2-deficiency decreased lung tumor growth and oncogenic Kras-mediated inflammatory response, and increased survival in a mouse model of lung cancer with conditional activation of mutant KRAS [21]. An increase in NOS2 and NOS3 expression is found in PDAC [9, 13], and genetic deficiency of endothelial NOS (NOS3) decreased the number of precursor lesions in mouse model of pancreatic cancer but failed to significantly enhance the survival of mice with PDAC [9]. As we found in our study, NOS2-deficiency enhanced survival in KPC mice with PDAC, and an increased NOS2 expression predicted poorer survival in patients with resected PDAC, which in our knowledge is the largest study so far to examine the role of NO in pancreatic cancer. Furthermore, these findings could be validated in the publically available datasets from independent cohorts of PDAC. Thus, NOS2 has a distinct role in the progression of PDAC and patient outcome. Nitric oxide is a signaling molecule, which induces a number of signaling pathways affecting multiple critical events such as apoptosis, proliferation, DNA repair, cell cycle arrest, and senescence. The resulting alterations in these events can generate conditions that are conducive to neoplastic changes. Additionally, NOS2/NO signaling is reported to mediate mutant KRAS-induced inflammation in lung cancer model [21], which is consistent with its role as an inflammatory mediator. Consistent with these known functions of NO, primary tumor cells from NKPC mice in our study were significantly less proliferative and showed reduced migratory and invasive properties, as compared to KPC mice. Additionally, an increased E-cadherin expression in NKPC tumor cells may be responsible for their reduced ability of migration and invasion as compared with KPC tumor cells. NO-dependent activation of c-Src results in the disruption of E-cadherin junction and enhanced breast cancer cell invasion [32]. In contrast, however, NO treatment enhanced E-cadherin expression in metastatic prostate cancer cells [33]. Pancreatic tumors in NKPC mice showed an overall better differentiation grade as compared with the tumors in KPC mice, indicating a reduced tumor severity in NKPC mice. Additionally, a significantly lower number of PanIN-1 lesions and absence of any carcinoma in NKPC mice as compared to the KPC mice, of which 30 percent (2 out of 6) showed the presence of carcinoma at an early age of 6 week, indicated an increased tumor-latency in NOS2-deficient NKPC mice. A highly inflammatory stroma with increased macrophage infiltration is reported in pancreatic tumors in KPC mice, which plays a role in the development and progression of PDAC [34]. In contrast, NKPC mice in our study showed a significantly decreased infiltration of macrophages, as determined by F4/80 expression, accompanied with a lower expression of CCL2/MCP-1 chemokine as compared with KPC tumors. CCL2 has been implicated in macrophage recruitment on the tumor site and enhanced cancer progression [35, 36]. Furthermore, an enhanced expression of CCL2 is associated with poorer survival in pancreatic cancer [19]. The presence of a lower expression of mir-21 in NKPC tumors as compared with tumors in KPC mice is consistent with the reports suggesting an association between increased NOS2 and mir-21 in lung cancer [21]. Although, an increased mir-21 expression is reported to be associated with inflammation-associated cancer, including that of pancreas, the exact mechanism of its regulation by inflammatory mediators, including NO, is not known.

Inactivation of FOXO tumor suppressor is implicated in the development and progression of human cancer. FOXO is involved in apoptosis, DNA repair, cell cycle arrest and immune regulation [25, 37–39]. FOXO is primarily regulated through PI3-kinase/AKT and ERK-mediated phosphorylation, leading to its ubiquitination and proteasomal degradation. Inhibition of PI3-kinase/AKT and ERK pathways led to the activation of FOXO, which resulted in the cell cycle arrest and apoptosis in pancreatic cancer [40]. Reduced phosphorylation of FOXO and increased apoptosis following the inhibition of PI3K/AKT and MAPK/ERK pathways resulted in the suppression of pancreatic tumor growth in orthotopic mouse model [41]. NO activates both ERK and PI3-kinase/AKT signaling. Consistently, NKPC tumors showed a lower pERK and pFOXO3 expression as compared to tumors in KPC mice. Moreover, treatment of pancreatic cancer cells with NO donor moderately enhanced pERK and pFOXO3 expression. These findings showed that NO phosphorylates/inactivates FOXO3 through the activation of ERK signaling. Recently, mir-21 is shown to inhibit nuclear retention of FOXO, which resulted in an increased cellular growth in PDAC [42]. However, the mechanism of mir-21 mediated regulation of FOXO is not known. Furthermore, in our study, a positive correlation between NOS2 and pFOXO3 in human PDAC provide clinical evidence of an association between NOS2/NO signaling and phosphorylation/inactivation of FOXO3.

Taken together, our findings in this study describes NOS2 as a candidate predictor of prognosis in early stage, resected PDAC patients, and provide proof-of-principle that targeting NOS2 may be a useful strategy to improve survival in this lethal malignancy.

## MATERIALS AND METHODS

### Human pancreatic ductal adenocarcinoma (PDAC) specimens

Fresh-frozen and paraffin embedded primary pancreatic tumor tissue from resected PDAC cases (*N* = 107) came from University of Maryland Medical System (UMMS), Baltimore, MD, through NCI-UMD resource contract and the University Medicine Gottingen, Gottingen, Germany. Demographic and clinical information, including age, sex, clinical staging, differentiation grade, resection margin status and survival from the time of diagnosis were available for each donor. Patients' characteristics are described in Supplementary Table S2. Use of the clinical specimens was reviewed and permitted by the NCI-Office of Human Subject Research (OHSR, Exempt# 4678) at the National Institutes of Health, Bethesda, MD.

### Mice

*LSL-Kras^G12D^; LSL-Trp53^R172H/+^; Pdx-1-Cre* (KPC) pancreatic cancer mouse model is generated through interbreeding of the *LSL-Kras^G12D^* and *LSL-Trp53^R172H/+^* mice with *Pdx-1-Cre* transgenic mice as described previously [43]. This leads to the activation of both *Kras^G12D^* and *Trp53^R172H/+^* alleles in the tissue progenitor cells of the pancreas and development of PDAC. To generate NOS2-deficient KPC mice, we first generated *LSL-Kras^G12D^; LSL-Trp53^R172H/+^; NOS2^+/−^* and *Pdx-1-Cre; NOS2^+/−^* strains of mice through interbreeding with *Nos2*^−*/*−^ mice. Then, by crossing of *LSL-Kras^G12D^; LSL-TP53^R172H/+^; NOS2^+/−^* and *Pdx-1-Cre; NOS2^+/−^* strains, we generated NOS2 wild type *LSL-Kras^G12D^;LSL-Trp53^R172H/+^; Pdx-1-Cre* (KPC) *and* NOS2*-*deficient *LSL-Kras^G12D^; LSL-Trp53^R172H/+^; NOS2*^−*/*−^*; Pdx-1-Cre* (NKPC) mice littermates. Mice were followed till they showed signs related to moribundity, previously described as indications preceding death in this mouse model of PDAC [43]. Mice were euthanized and a complete necropsy was performed on each mouse. Tissue samples were paraffin fixed and snap frozen for further analysis.

### Pathological characterization of tumor severity

Overall tumor differentiation was determined by the grade of highest percentage of tumors on each slide. Respectively, grade 1 represents mostly PanINs or scattered early well-differentiated tumors, grade 2 tumors are well differentiated (minimal to no EMT), grade 3 tumors are moderately differentiated (more glandular than EMT), grade 4 tumors are poorly differentiated (mostly EMT, but may still have a few glands), grade 5 tumors are sarcomatoid (all EMT).

### Mouse primary tumor cell lines

Mouse primary tumor cells were isolated as previously described [43] Isolated mouse primary tumor cell lines were maintained in DMEM-F12 (Life Technologies, Grand Island, NY) medium containing 10% FBS. Assays were conducted within 8 passages of the cell lines.

### Xcelligence cell proliferation

Cell proliferation was measured using E-plates (ACEA, San Diego, CA). Cell index was recorded by Xcelligence system (ACEA, San Diego, CA) as indicator of cell numbers. Mouse primary tumor cells from KPC or NKPC mice were digested into single cell suspension by trypsin, and seeded into E-plate at a concentration of 500 cells/well. E-plates were loaded onto Xcelligence system and cell index were recorded every 30 min for 80 hours for proliferation assay. Each curve represents the average of quadruplicated wells.

### Mouse cytokine array

Mouse cytokine array was purchased from R&D systems (Minneapolis, MN). Briefly, KPC and NKPC pancreas tissue homogenate were prepared as described above. Thirty micrograms of tissue homogenate from each of the 10 KPC and 10 NKPC mice were used to make a total 300 ug protein mixture for each group. The volumes of protein mixtures were adjusted as described by manufacturer's instruction. The following steps were similar to western blot. Visible spots representing different cytokines were quantified by using Quantity One software (Bio-rad, Hercules, CA).

### Statistical analysis

Kaplan-Meier analysis was performed, using Graphpad Prism 6.0, to evaluate the differences in survival probability in patients with pancreatic cancer, and also in mouse model. For survival analysis NOS2 gene expression was dichotomized as high and low groups based on the median value. Comparisons of gene and microRNA expression were done by unpaired *T*-test. Comparisons of IHC staining were conducted using Mann-Whitney Test. The correlation between NOS2 and pFOXO was determined by Pearson correlation. A *p*-value less than 0.05 was considered significant. Other materials and methods are explained in the supplementary information.

## SUPPLEMENTARY MATERIAL FIGURES AND TABLES


