# Supplementary material for: Inducible nitric oxide synthase enhances disease aggressiveness in pancreatic cancer
Source: Oncotarget. 2016 Jun 29;7(33):52993–3004. doi: 10.18632/oncotarget.10323 (PMC5288163; doi:10.18632/oncotarget.10323)
Supplement: Supplementary file 1 [file oncotarget-07-52993-s001.pdf]

Inducible nitric oxide synthase enhances disease aggressiveness in pancreatic cancer

Supplementary Materials

SUPPLEMENTARY MATERIALS AND METHODS

RNA Extraction and qPCR

Total RNA was extracted from frozen tumor samples using a standard Trizol protocol (Invitrogen, Carlsbad, CA). RNA from cultured cells was isolated using the Total RNA extraction kit (Norgenbiotek,Thorold, Canada) according to the manufacturer's protocol. RNA quality was evaluated using Agilent 2100 Bioanalyzer (Agilent Technologies, Wilmington, DE). RNA was reverse-transcribed using Multi Scribe reverse transcriptase (Life Technology, Foster City, CA). Gene-expression levels of NOS2 and miR-21 were detected using Taqman probes (Life Technologies, Grand Island, NY).

Immunohistochemistry and antibodies

5 μm-thick paraffin-fixed tumors sections from resected PDAC cases or mouse PDAC tumors were deparaffinized, hydrated and incubated with antibodies listed below or isotype-matched negative controls, followed by incubation with HRP-conjugated secondary antibodies and DAB chromogen detection system (Dako Envision System, Dako, Carpinter, CA). For computer-assisted quantitation, the painted (immunostained) area of each field was calculated using MetaMorph software (Molecular Devices Corp., Sunnyvale, CA) [44]. Anti-NOS2 antibody (160862, Cayman Chemicals, Ann Arbor, MI), anti pFOXO3 (ab154786, Abcam, Cambridge, MA) cleaved Caspase3 antibodies (ab2302, Abcam, Cambridge, MA), anti p-ERK antibody (4370L, Cell Signaling, Danvers, MA), anti-F4/80 antibody (MF448000, Thermo Fisher Scientific, Waltham, MA), were obtained commercially.

Human PDAC cell lines

Human pancreatic cancer cell lines, Capan2 and SU.86.86 were purchased from the American Type Culture Collection, ATCC (Rockville, MD) and authenticated by short tandem repeat (STR) analysis. Capan2 cells were grown in McCoy's 5A (modified) medium and SU.86.86 cells were grown in RPMI 1640, supplemented with 10% fetal bovine serum, penicillin-streptomycin (50 IU/ml and 50 mg/ml, respectively) (Sigma Aldrich, St. Louis, MO) and 2 mM L-glutamine in a humidified incubator containing 5% CO2 at 37°C. All products for cell culture were purchased from Life Technologies (Grand Island, NY).

Wound healing

Twenty-four hours prior to assay, KPC and NKPC primary tumor cells were seeded into 6-well plates to reach a final confluency of ~90%. Wounds were made by scratching the bottom of the plates with pipette tips. Pictures of the same region of the wounds were taken at 0 and 24 hours.

Transwell migration and invasion assay

Cell migration and invasion were measured by BD chamber migration and invasion system as described by manufacturer (BD Biosciences, San Jose, CA). KPC and NKPC primary tumor cells were made into single-cell suspension in DMEM-F12 medium by trypsin treatment and applied onto upper chamber, at a density of 2×10^5 cells/well. The complete medium (DMEM-F12 with 10% FBS) was loaded into lower chamber. Twenty-two hours after adding the cells onto upper chambers, the upper chambers were taken out and washed with PBS, followed by fixation in 100% methanol and staining with 0.1% crystal violate. Experiments were repeated at least 3 times.

Western blotting

Cells were washed in PBS and proteins were extracted with RIPA buffer (Life Technologies, Grand Island, NY). Electrophoresis was carried out on 4–20% SDS-PAGE gels (Life Technologies, Grand Island, NY), and then transferred on to a nitrocellulose membrane (Life Technologies, Grand Island, NY). To block nonspecific binding, the membrane was incubated for 60 min with 5% non-fat milk in PBST at room temperature. The membrane was then washed with PBST and incubated overnight with antibodies indicated and anti β-actin antibody (Sigma Aldrich: St. Louis, MO) as endogenous control in PBST. Secondary anti-mouse or -rabbit antibodies with HRP were purchased from Santa Cruz (Santa Cruz, CA). Specific protein was visualized using a Super-Signal protein detection kit (Pierce, Rockford, IL) according to manufacturer's instructions.

Immuno-fluorescence

A standard immunofluorescent method was used for staining. Cells were seeded into 6-well plates one day prior to the experiment with a glass coverslip placed in each well in advance. Cells were allowed to attach and grow under normal culture condition as mentioned earlier for 24 hours before being harvested. Prior to staining, coverslips with cells attached were washed with DPBS (Invitrogen, Carlsbad, CA) for 3 times and then fixed with 4% paraformaldehyde for 10 min. After fixation, cells were washed again with DPBS for 3 times and stained with 1:100 diluted Alexa Fluor555 pahlloidin (Cell Signaling Technology) for 30 min, and then washed with DPBS for 3 times. To mount the stained cover slip, a drop of DAPI (Sigma-Aldrich, St. Louis, MO) was dropped onto a slide and cover slips were placed onto DAPI drop. Actin filaments were observed under Zeiss fluorescent microscope (Carl Zeiss Inc, Thornwood, NY).

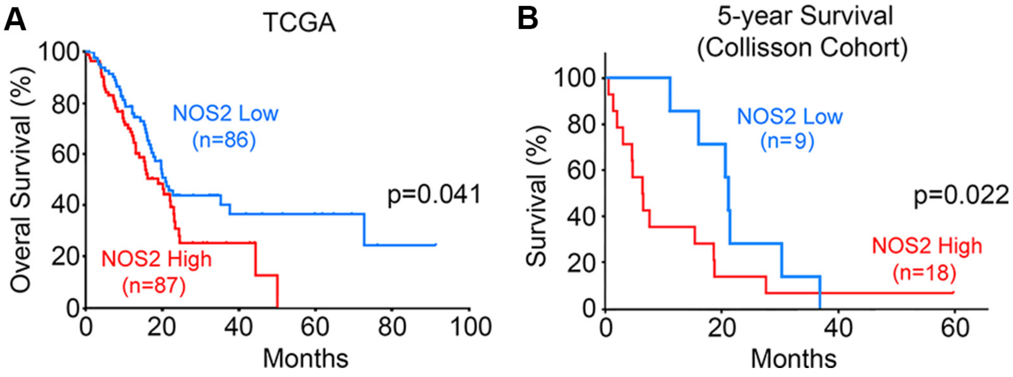

Supplementary Figure S1: Higher NOS2 expression is associated with poorer survival in publicly available gene-expression data sets from patients with PDAC. (**A**) A higher expression of NOS2 is also associated with poorer survival in TCGA cohorts (N = 173) and (**B**) Collisson cohort (N = 27) [14] dataset in Oncomine database.

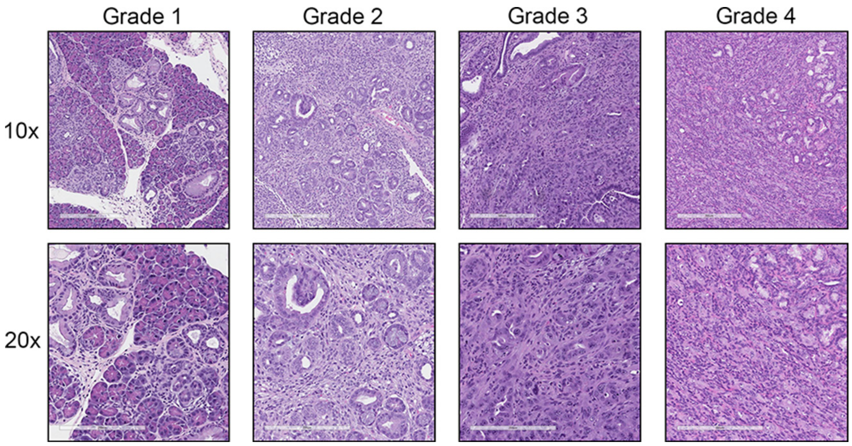

Supplementary Figure S2: Representative H/E pictures showing tumor differentiation Grade 1–4 in the KPC mouse model of pancreatic cancer.

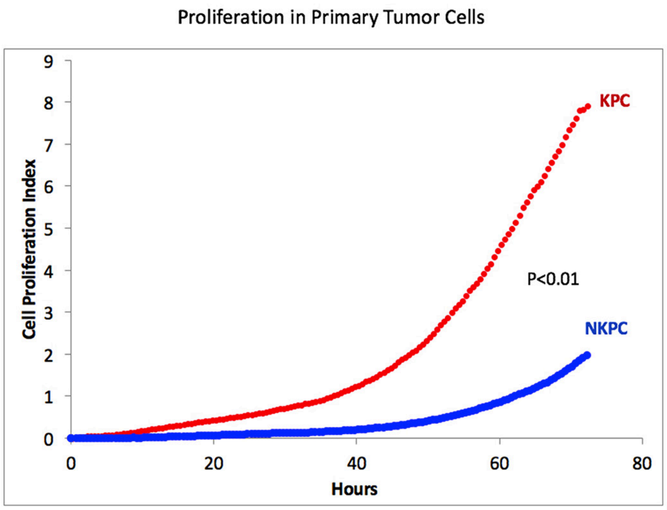

Supplementary Figure S3: Primary tumor cells isolated from additional KPC mouse showed enhanced proliferation as compared with NKPC mouse.

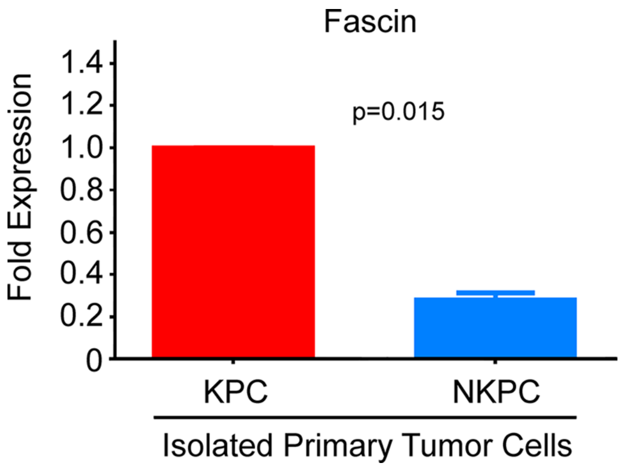

Supplementary Figure S4: NOS2-deficiency decreased Fascin expression in primary tumor cells from NKPC mice. Fascin expression was determined by qRT-PCR in KPC and NKPC primary tumor cells. Data represents 3 biological replicates from primary tumor cells from each mouse in either NKPC or KPC group.

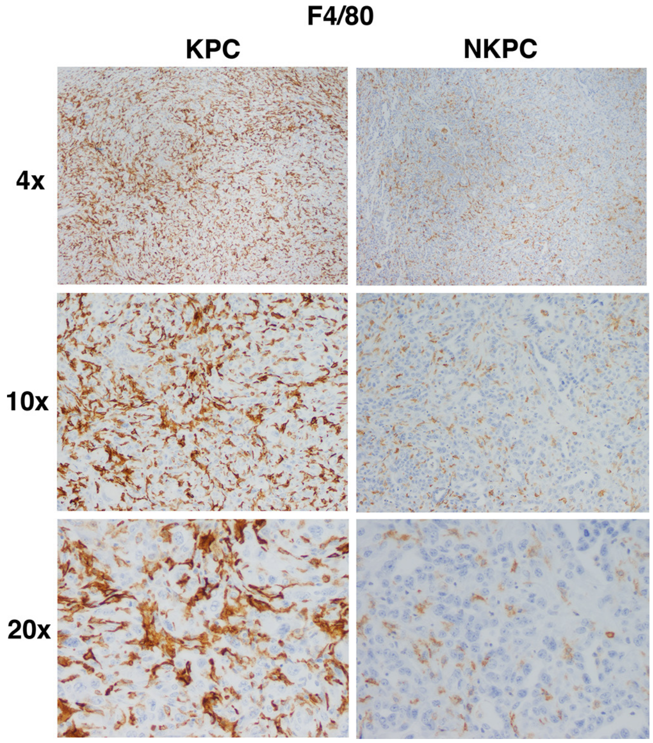

Supplementary Figure S5: Additional pictures showing an increased F4/80 expression in tumors from KPC mice as compared to NKPC mice.

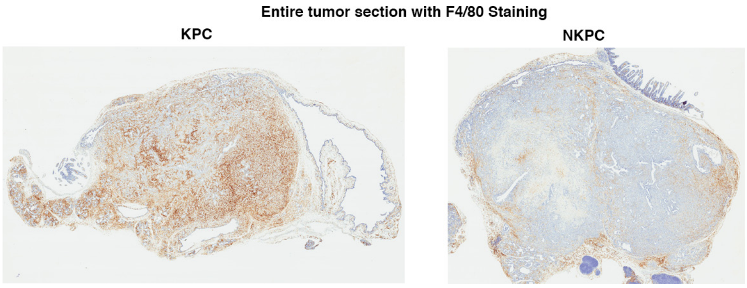

Supplementary Figure S6: Additional pictures showing an increased F4/80 expression in tumors from KPC mice as compared to NKPC mice.

Supplementary Table S1: Cox proportional hazard analysis

Variables (comparison/referent)	Univariable Analysis		Multivariable Analysis	
	HR (95% CI)	*P*	Hazard Ratio	*P*
NOS2 (High/Low)	1.73 (1.09–2.75)	***0.020***	1.75 (1.10–2.79)	***0.018***
Resection Margin (R1/R0)	1.27 (0.80–2.02)	0.305	1.22 (0.76–1.96)	0.407
Grading (G3-4/G1-2)	1.36 (0.86–12.15)	0.194	1.21 (0.75–1.95)	0.444
Stage(II/I)	1.6 (0.94–2.74)	0.085	1.61 (0.93–2.79)	0.087

Supplementary Table S2: Characteristics of Patients (*N* = 107)

Age at enrollments (y)	
Mean (SD)	67.1 (9.2)
Range	38–87
Gender, no. (%)	
Male	24 (48.98)
Female	25 (51.02)
Stage group, no. (%)	
IA	3 (2.80)
IB	10 (9.35)
IIA	17 (15.89)
IIB	77 (71.96)
Grading, no. (%)	
G1	5 (4.67)
G2	58 (54.21)
G3	41 (38.32)
G4	3 (2.80)
Resection margin, no. (%)	
R0	49 (45.79)
R1	58 (54.21)
